# The imPAct of Trimetazidine on MicrOcirculation after Stenting for stable coronary artery disease (PATMOS study)

**DOI:** 10.3389/fcvm.2023.1112198

**Published:** 2023-06-30

**Authors:** Ivan Ilic, Stefan Timcic, Maja Milosevic, Srdjan Boskovic, Natalija Odanovic, Matija Furtula, Milan Dobric, Srdjan Aleksandric, Petar Otasevic

**Affiliations:** ^1^ Cardiology Clinic, Institute for Cardiovascular Diseases Dedinje, Belgrade, Serbia; ^2^ Faculty of Medicine, University of Belgrade, Belgrade, Serbia; ^3^ Cardiology Clinic, University Clinical Center of Serbia, Belgrade, Serbia

**Keywords:** Trimetazidine (TMZ), index of microcirculatory resistance (IMR), PCI-percutaneous coronary intervention, chronic coronary artery disease, coronary flow

## Abstract

**Background:**

Myocardial ischemia is caused by epicardial coronary artery stenosis or atherosclerotic disease affecting microcirculation. Trimetazidine (TMZ), promotes glucose oxidation which optimizes cellular energy processes in ischemic conditions. Small studies demonstrated protective effects of TMZ in terms of reducing myocardial injury after percutaneous coronary intervention (PCI), its effect on microcirculation using contemporary investigative methods has not been studied. The aim of the study was to examine effects of trimetazidine, given before elective PCI, on microcirculation using invasively measured index of microcirculatory resistance (IMR).

**Methods:**

This was prospective, single blinded, randomized study performed in a single university hospital. It included consecutive patients with an indication for PCI of a single, *de novo*, native coronary artery lesion. Patients were randomly assigned to receive either TMZ plus standard therapy (TMZ group) or just standard therapy. Coronary physiology indices fractional flow reserve (FFR), coronary flow reserve (CFR) and index of microcirculatory resistance (IMR) were measured before and after PCI using coronary pressure wire.

**Results:**

We randomized 71 patients with similar clinical characteristics and risk profile, previous medications and coronary angiograms. Patientshad similar values of Pd/Pa, FFR and CFR prior to PCI procedure. After PCI, FFR values were higher in TMZ group, while IMR values were lower in this group respectively (FFR TMZ + 0.89 ± 0.05 vs. TMZ – 0.85 ± 0.06, *p* = 0.007; CFR TMZ + 2.1 ± 0.8 vs. TMZ- 2.3 ± 1.3, *p* = 0.469; IMR TMZ + 18 ± 9 vs. TMZ- 24 ± 12, *p* = 0.028). In two-way repeated measures ANOVA PCI was associated with change in FFR values (TMZ *p* = 0.050; PCI *p* < 0.001; *p* for interaction 0.577) and TMZ with change in IMR values (TMZ *p* = 0.034, PCI *p* = 0.129, *p* for interaction 0.344).

**Conclusion:**

Adding trimetazidine on top of medical treatment prior to elective PCI reduces microvascular dysfunction by lowering postprocedural IMR values when compared to standard therapy alone.

## Background

The myocardial ischemia is caused by stenosis of the epicardial coronary artery or atherosclerotic disease affecting small coronary vessels—microcirculation. Not until recently, the importance of the coronary microcirculation in causing symptoms and signs of ischemia has been appreciated. Microcirculatory dysfunction has an important effect in determining patient outcomes (1–3). Recently a term INOCA (ischemia with no obstructive coronary arteries) was introduced, in order to group large number of patients having symptoms of angina, but not having significant stenoses of coronary arteries (4). At the molecular level ischemia is caused by cellular energy disbalance resulting in deficit of adenosine−5′-triphosphate (5). Although the mechanism of ischemia is well known, there is a paucity of trials addressing tailored medical treatment for these patients. Trimetazidine (TMZ), an anti-ischemic drug, exhibits its action by inhibiting ß-oxidation of fatty acids in cardiomyocites, thus promoting glucose oxidation which optimizes cellular energy processes in ischemic conditions. TMZ maintains the necessary level of ATP in the cell, protecting it from acidosis and calcium overload (6, 7). Several studies confirmed the effects of TMZ in patients with stable angina and it has been used as second line drug for chronic coronary syndrome (8). Although small studies demonstrated protective effects of TMZ in terms of reducing myocardial injury after percutaneous coronary intervention (PCI), its effect on microcirculation using contemporary investigative methods has not been studied (9, 10).

### Aim

The aim of the study was to examine effects of trimetazidine, given before elective PCI, on microcirculation using invasively measured index of microcirculatory resistance (IMR).

## Methods

This was prospective, single blinded, randomized study performed in a single university hospital. The study included consecutive patients with stable angina or anginal equivalent with positive non-invasive stress test and an indication for PCI of a single, *de novo*, native coronary artery lesion classified as AHA/ACC class A/B1 with diameter stenosis (DS) visually estimated to be greater than 70%. The patients were scheduled for PCI based on coronary angiograms that were done previously. In order to avoid additional, unnecessary procedures further increasing the risk to the patients, the study subjects were selected among the ones waiting for staged procedures or the ones whose angiograms were submitted for review and treatment after coronary angiography was done in another institution. Exclusion criteria were acute coronary syndrome (ACS) less than 30 days before PCI, decreased left ventricular systolic function (LVEF) of less than 35%, previous PCI in treated artery, a history of previous myocardial infarction in the territory supplied by the treated coronary artery, existence of the collateral circulation to another coronary artery supplied by the treated vessel, chronic total occlusion, significant bifurcation lesion defined as coronary artery lesion at the site of bifurcation where the side branch is greater than 2 mm and is perceived important by the operator, previous surgical revascularization, significant renal dysfunction with estimated glomerular filtration rate (eGFR) of less than 60 ml/min. Patients were randomly assigned using computer based algorithm to receive either TMZ plus standard therapy (TMZ group) or just standard therapy prior to scheduled PCI. Based on previous studies on pharmacokinetics of TMZ and animal and clinical studies that explored immediate effects of oral loading of TMZ, patients were to receive standard dose of TMZ slow-release tablets of 35 mg twice a day at least 48 h prior hospital admission for intervention. The patients that were TMZ naïve were given oral loading dose of 70 mg at least 48 h prior to PCI procedure, then continued 35 mg twice a day (11–13). Patients were contacted prior to admission for PCI and after obtaining verbal consent to participate in the trial they were asked to start TMZ or continue their previous therapy based on assigned randomization and TMZ status in already prescribed medications. On admission, the medication status was checked and confirmed patients’ compliance to the prescribed treatment and study group.

The participants gave written informed consent to take part in the study and were asked to fill out the predefined questionnaire. The PCI operator was not aware of patient’s study allocation to TMZ or not. The study was conducted in accordance with Helsinki declaration and was approved by institutional Ethics committee. The study protocol was registered at clinicaltrials.gov (NCT 02107144).

PCI procedure was prepared according to hospital protocol, patients received dual antiplatelet therapy prior to PCI consisting of aspirin and one of P2Y12 inhibitors (clopidogrel, ticagrelor or prasugrel) depending on whether they previously had an ACS or not. PCI procedure was done according to study protocol which mandated predilatation of the lesion and no direct stent implantation to avoid procedural discrepancies which can affect invasive physiology measurements. If postdilatation was required or another stent needed to treat dissection invasive physiology measurements were done prior to continuation of PCI procedure. Quantitative coronary angiography (QCA) measurements were obtained by acquiring the following data: reference vessel diameter (RVD), minimal lumen diameter (MLD) and diameter stenosis (DS) prior to PCI and residual diameter stenosis (RDS) after the intervention was done.

Coronary physiology indices fractional flow reserve (FFR), coronary flow reserve (CFR) and index of microcirculatory resistance (IMR) were measured before and after PCI using coronary pressure—/thermistor—tipped wire (Pressure Wire X, Abbott Vascular, Plymouth, MN, US). Maximal hyperaemia was achieved using intravenous infusion of adenosine (140 mcg/kg/min) using infusion pump. Simultaneous recordings of mean aortic pressure (guiding catheter, P*a*) and mean distal coronary pressure (distal pressure sensor, P*d*) were obtained at baseline and during maximal hyperaemia. FFR was calculated by dividing the mean distal coronary pressure (Pd) by the mean aortic pressure (Pa) during maximal hyperemia. CFR was calculated as a ratio of mean transit time of 3 ml of normal saline at basal condition (T_bas_) and after inducing hyperemia (T_hyp_) (14, 15). The IMR was calculated using the following equation: IMR = Pa × T_hyp_ [(Pd-Pw)/(Pa-Pw)], where Pw is the coronary wedge pressure. Pw was measured as the distal coronary pressure Pd (from the distal pressure and temperature sensor) during complete balloon occlusion of the vessel with the angioplasty balloon during PCI. The lesion was predilated using semi- compliant or non-compliant balloon sized 0.75–1.0 ratio to reference vessel diameter. The measurements were done using dedicated Coroventis CoroFlow cardiovascular software (Coroventis AB, Uppsala, Sweden). Patients’ data were obtained and recorded in previously formed database.

Patients were discharged after 24 h if postprocedural course was uneventful. Levels of postprocedural markers of myocardial necrosis CK and CK-MB were collected at 6, 12 and 24 h post PCI. The peak values were recorded in patients’ database.

### Statistical analysis

Continuous data were summarized as the means ± SD. Categorical data were summarized as counts and percentages. Unpaired *t*-test was used for comparing the continuous variables, if the distribution was normal, and Mann–Whitney *U* test if the distribution was not normal. Chi-squared test and Fisher’s exact test were used for categorical variables. Two-way repeated measures ANOVA was used for comparing the effects of TMZ and PCI on variables that represent invasive physiology parameters. We defined PCI as within-subject factor (prior to PCI vs. after PCI) and drug treatment (TMZ+ vs. TMZ−) as a between-subject factor for performing ANOVA. Tukey statistical test was used for *post hoc* analysis. A *p*-value of *p* < 0.05 was considered statistically significant. All statistical analyses were performed using IBM SPSS Statistics 19.0 statistical software (IBM Armonk, New York, NY, US).

Based on the previous studies it was estimated that study sample size needed for expected difference in IMR values of 9 to be obtained between study groups (power 80%) is around 40 patients (16, 17).

## Results

**Figure 1:**
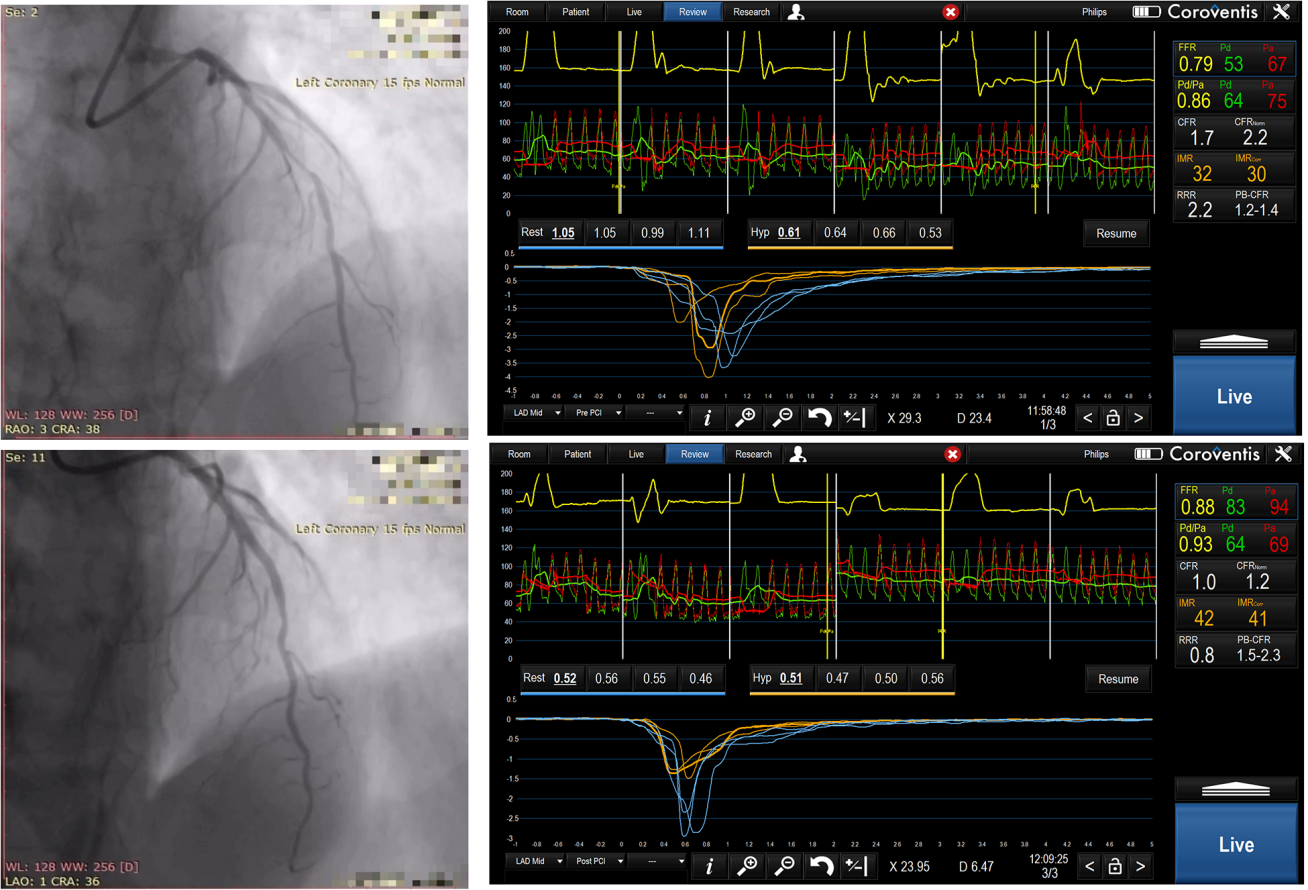
Sixty-two years old male, randomized to TMZ -, predilatation with 2.5 × 15 mm non compliant balloon, then drug eluting stent 3.0 × 24 mm was implanted in mid LAD. Angiographic images and physiology measurements before and after PCI were shown, please note high IMR and low CFR values.

The study was done from February 2021 until September 2022, at Dedinje Cardiovascular Institute high volume university PCI center, with 24/7 interventional cardiology service, and included 71 patients that were randomized in the study. Initially 91 patients were evaluated for the study, but 20 patients (21.9%), prior to planned PCI, had initial FFR values above 0.80 in the artery to be treated and were denied PCI procedure. The clinical characteristics of study groups were well balanced with risk profile typical for transitional country. There was numerical difference in number of patients having peripheral arterial disease between the study groups that did not reach statistical significance (Table 1). Patients’ groups were well balanced regarding presence of microvascular dysfunction prior to PCI procedure defined as CFR less than 2.0 and IMR greater than 25 [TMZ + 8/36 (22.2%) vs. TMZ – 6/35 (17.1%); *p* = 0.178]. All patients received dual antiplatelet therapy (DAPT) consisting of aspirin and one of the available P2Y12 inhibitors (clopidogrel or ticagrelor). Also, the other medications given to the patients were well balanced. It’s worth noting that patients treated with TMZ were less frequently prescribed with an angiotensin converting enzyme (ACE) inhibitor (Table 2). There was no difference between study groups regarding presence of multivessel disease and specific coronary artery treated with PCI. Also, QCA measurements were similar between patients’ groups. The total length and diameter of the stents implanted were similar in patients’ groups. There was no difference between study groups regarding need for postdilatation. Coronary dissection occurred in three patients in TMZ + group and in four patients in TMZ—groups and all of them were treated with another stent implantation. None of these dissections was greater than type B, so it didn’t affect coronary flow, nor it caused significant lumen compromise, so we perceived that it didn’t affect coronary physiology measurements. Thrombolysis in myocardial infarction (TIMI) III flow was achieved in every patient after PCI (Table 2 and Figure 1).

**Table 1:** Clinical characteristics of patients randomized to trimetazidine or standard therapy prior to PCI.

Variable	TMZ + *n* = 36	TMZ − *n* = 35	*p*-value
Age (years)	64 ± 10	65 ± 9	0.662
Males *n* (%)	28 (77.8)	27 (77.1)	0.587
Heredity *n* (%)	9 (25)	9 (25.7)	0.580
Hypertension *n* (%)	32 (88.9)	34 (97.1)	0.187
Diabetes mellitus *n* (%)	13 (36.1)	2 (42.8)	0.368
Dyslipidemia *n* (%)	20 (55.5)	17 (48.5)	0.493
PAD *n* (%)	2 (5.5)	7 (20.0)	0.085
Current smoking *n* (%)	21 (58.3)	24 (68.5)	0.258
Previous MI *n* (%)	18 (50)	17 (48.5)	0.547
Previous CVI *n* (%)	1 (2.8)	2 (5.7)	0.489
COPD *n* (%)	1 (2.8)	1 (2.8)	0.746
BMI (kg/m^2^)	27.8 ± 4.2	28.6 ± 4.8	0.447
LVEF (%)	48.3 ± 10.7	51.1 ± 7.8	0.214

BMI, body mass index; COPD, chronic obstructive pulmonary disease; CVI, cerebrovascular insult; LVEF, left ventricular ejection fraction; MI, myocardial infarction; PAD, peripheral arterial disease.

**Table 2:** Characteristics of PCI procedure in patients randomized to trimetazidine or standard therapy prior to PCI.

Variable	TMZ + *n* = 36	TMZ − *n* = 35	*p*-value
Coronary artery treated			0.662
LAD *n* (%)	15 (41.7)	17 (48.5)	—
Cx-OM *n* (%)	12 (33.3)	11 (31.4)	—
RCA *n* (%)	9 (25.0)	7 (20.0)	—
Multivessel disease *n* (%)	16 (44.4)	12 (34.3)	0.264
RVD before PCI (mm)	2.8 ± 0.2	2.9 ± 0.4	0.373
DS before PCI (%)	71 ± 8	69 ± 9	0.425
MLD before PCI (mm)	1.2 ± 0.3	1.4 ± 0.3	0.136
Residual DS after PCI (%)	13 ± 9	17 ± 12	0.089
Total stent length (mm)	21.5 ± 4.8	22.7 ± 6.2	0.421
Stent diameter (mm)	3.2 ± 0.4	3.1 ± 0.4	0.588
Postdilatation *n* (%)	15 (41.7)	13 (37.1)	0.753
Clopidogrel *n* (%)	29 (80.6)	30 (85.7)	0.202
Ticagrelor *n* (%)	7 (19.4)	5 (14.3)	0.274
Beta blockers *n* (%)	35 (97.2)	31 (88.5)	0.170
ACE inhibitor *n* (%)	27 (75.0)	32 (91.4)	0.062
Statin *n* (%)	36 (100)	35 (100)	0.746

ACE, angiotensin converting enzyme; Cx, circumflex artery; DS, diameter stenosis; LAD, left anterior descending; MLD, minimal lumen diameter; OM, obtuse marginal; RCA, right coronary artery; RVD, reference vessel diameter.

**Figure 2:**
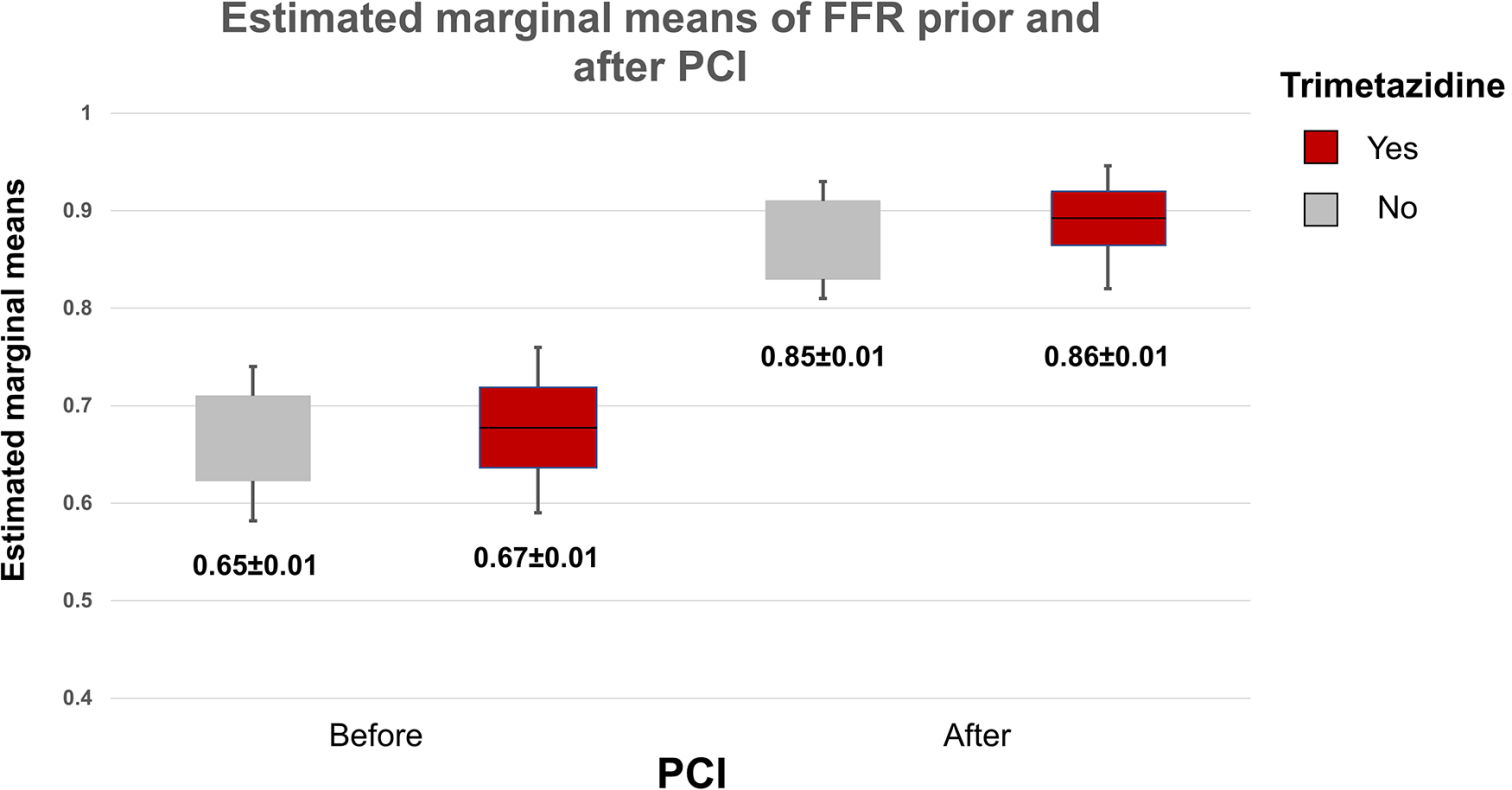
Plot demonstrates two-way repeated measures ANOVA statistics of influence of TMZ and PCI on estimated interaction between the drug and the procedure regarding effects on FFR values (box and whiskers diagram of estimated marginal means of FFR before and after PCI).

**Figure 3:**
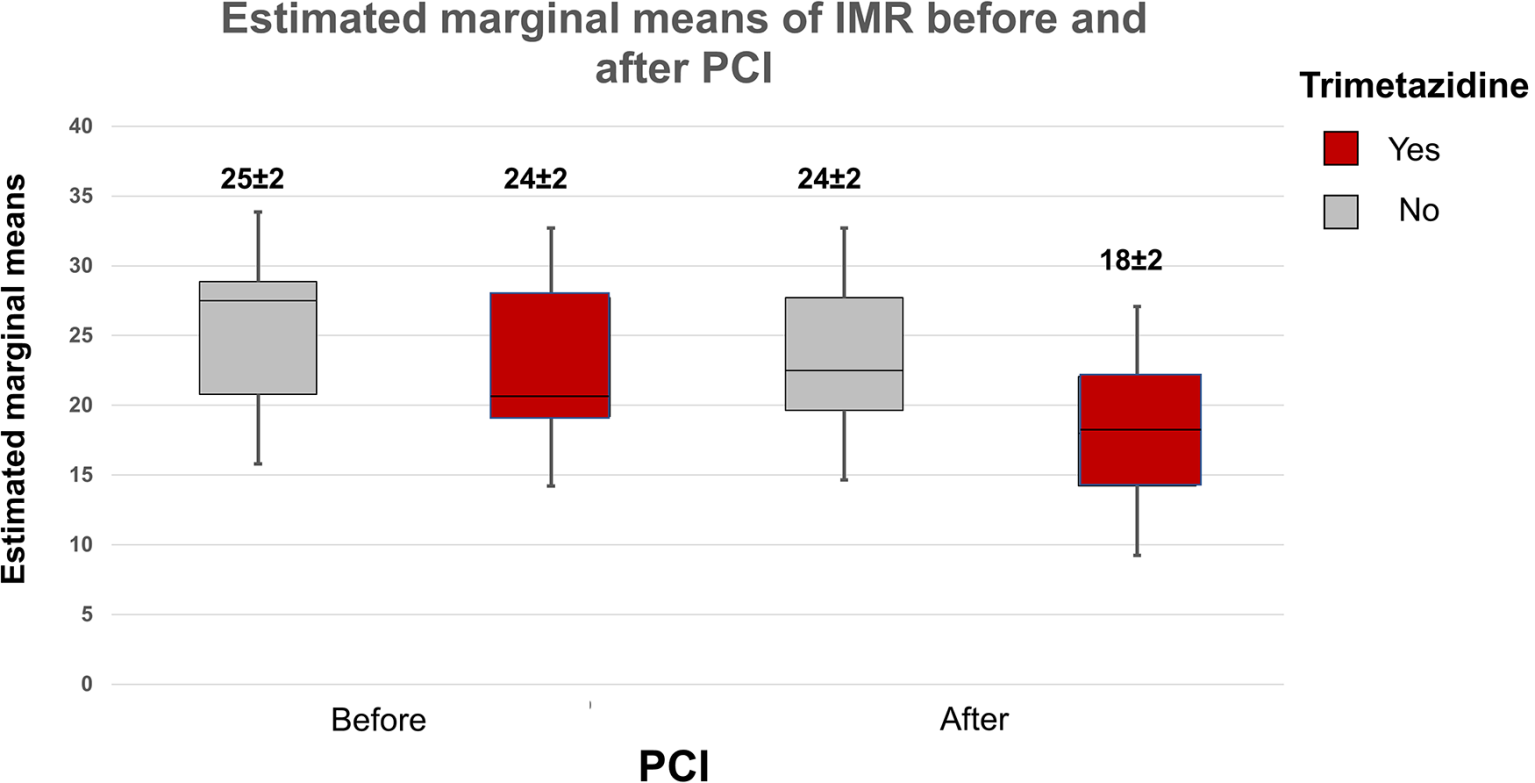
Plot demonstrates two-way repeated measures ANOVA statistics of influence of TMZ and PCI on estimated interaction between the drug and the procedure regarding effects on IMR values (box and whiskers diagram of estimated marginal means of IMR before and after PCI).

Postprocedural peak values of CK and CK—MB were similar in the study groups (CK TMZ+ 149 ± 127 IU vs. TMZ− 132 ± 90 IU; *p* = 0.567; CK-MB TMZ+ 19 ± 10 IU vs. TMZ− 17 ± 7 IU; *p* = 0.420). The invasive physiology procedures and PCIs were completed uneventfully. Patients in study groups had similar values of Pd/Pa, FFR and CFR prior to PCI procedure. Wedge pressure values were lower in TMZ group. After PCI Pd/Pa and CFR values were similar in the study groups, but FFR values were higher in TMZ group, while IMR values were lower in this group (Table 3). Using two-way repeated measures ANOVA we did not demonstrate independent effect of TMZ nor PCI procedure on CFR (TMZ F statistic 0.020, dF 1 *p* = 0.888; PCI F statistic 2.651, dF 1, *p* = 0.106; *p* for interaction 0.345), while both had effect on FFR (TMZ, F statistic 3.909, dF 1 *p* = 0.050; PCI F statistc 252.243, dF 1, *p* < 0.001; *p* for interaction 0.577) and values of IMR were affected dominantly by TMZ compared to PCI (TMZ F statistic 7.453, dF 1, *p* = 0.034, PCI F statistic 2.651, dF 1 *p* = 0.129, *p* for interaction 0.344) and Pd/Pa values were influenced mostly by PCI without significant interaction with TMZ given (TMZ F statistic 0.044, dF 1, *p* = 0.834, PCI F statistic 131.186, dF 1, *p* < 0.001; *p* for interaction 0.373) (Figures 2, 3).

**Table 3:** Characteristics of invasive physiology measurements in patients randomized to trimetazidine or standard therapy.

Variable	TMZ + *n* = 36	TMZ − *n* = 35	*p*-value
Pd/Pa prior to PCI	0.75 ± 0.17	0.78 ± 0.16	0.586
Pd prior to PCI (mmHg)	53 ± 17	57 ± 13	0.311
FFR prior to PCI	0.67 ± 0.11	0.65 ± 0.09	0.430
*T*_mn_ hyperemia before PCI (s)	0.44 ± 0.26	0.43 ± 0.28	0.854
CFR prior to PCI	2.8 ± 1.3	2.5 ± 1.9	0.518
IMR prior to PCI	23 ± 13	25 ± 13	0.594
Wedge pressure (mmHg)	10 ± 7	14 ± 5	0.032
Pd/Pa after PCI	0.91 ± 0.04	0.88 ± 0.07	0.370
Pd after PCI (mmHg)	69 ± 15	66 ± 17	0.422
*T*_mn_ hyperaemia after PCI (s)	0.27 ± 0.19	0.36 ± 0.28	0.225
FFR after PCI	0.89 ± 0.05	0.85 ± 0.06	0.007
CFR after PCI	2.1 ± 0.8	2.3 ± 1.3	0.469
IMR after PCI	18 ± 9	24 ± 12	0.028

CFR, coronary flow reserve; FFR, fractional flow reserve; IMR, index of microcirculatory resistance anterior descending; Pa, arterial pressure; Pd, distal pressure; *T*_mn_, transit mean time; RCA, right coronary artery; OM, obtuse marginal.

## Discussion

Successful PCI has been traditionally determined by optimal angiographic result. Despite this, between 20%–40% of patients after PCI experience angina (18). Although mechanisms of post PCI angina occurrence differ, this is at least partly due to microvascular dysfunction and myocardial injury after PCI (19). A series of functional assessments of stented arteries such as FFR, CFR and IMR have, therefore, been used for functional assessment of microcirculation after successful PCI (17–21). Based on intracoronary physiology assessment, recent studies reported that suboptimal values were found in 10%–36% of patients (22–24).

In our randomized, controlled, study conducted in patients with stable coronary artery disease, we found that pre-treatment with TMZ might improve coronary microvascular function and prevents the occurrence of microvascular impairment related to PCI. Our study revealed significantly higher post PCI FFR and significant decrease in IMR after PCI in patients treated with additional TMZ compared to standard medical treatment. TMZ pre-treatment did not influence the values of measured CFR. There was no significant interaction between effects of given TMZ and PCI procedure regarding indices of coronary physiology (CFR, FFR, IMR). As expected, PCI procedure had strong influence on Pd/Pa and FFR that was not demonstrated with TMZ therapy, while TMZ influenced IMR values which cannot be said for PCI procedure. Also, there was no difference between the study groups regarding postprocedural markers of myocardial necrosis. Effects of TMZ on microcirculation are complex. In ischemic myocardium and especially in microvascular angina it primarily decreases cardiac fatty acid β-oxidation, while increasing pyruvate dehydrogenase and thus stimulating the rate of mitochondrial ATP production (25). This results in increased production of vasodilating agent NO, decrease in reactive oxygen species (ROS) and reduced apoptosis of myocardial cells. Although its effect is mostly limited to myocardial cells, it can promote vasodilatation thus reducing ischemia caused myocardial injury and can affect platelet aggregation (12, 26, 27). It has been shown that TMZ, given prior to PCI in patients with unstable angina who underwent PCI reduced risk of postprocedural CK-MB and troponin elevation. Several trials addressed the use TMZ influence on myocardial injury and although results were variable, in a meta-analysis of 14 trials pre-treatment with TMZ was associated with reduced levels of high-sensitive troponin after elective PCI but did not affect the levels of CK-MB (9, 10). In our study we’ve included only patients with simple lesions (type A/B1) in order to reduce the effect of the procedure on microcirculation, thus reducing the potential of distal embolization and myocardial injury. The lack of protective effect of TMZ on release of CK-MB in our study might be caused by relatively simple PCI procedures that did not cause prolonged ischemia and the use of CK-MB as a marker of myocardial necrosis that is less sensitive to subtle ischemia compared to high sensitive troponin.

There was a divergent change in IMR after PCI related to FFR values before and after PCI. The relationship between these two variables may not be linear and direct. It depends on mean transit time (*T*_mn_ hyperemia) and wedge pressure. Since we measured wedge pressure only once during balloon predilatation before stent implantation, this could be the cause for obtaining different values of IMR after PCI after applying the formula for IMR calculation (Pw TMZ + group 10 ± 7 mmHg vs. Pw TMZ—group 14 ± 5 mmHg; *p* = 0.032). Although the values of Pd and Pa are included in IMR calculation equation, it is also influenced by the values of *T*_mn_ in hyperemia as well as value of wedge pressure. In the study by Murai T., et al. there was a correlation between values of post PCI FFR and IMR (*R*^2 ^= 0.134, *p* < 0.001) with relatively low value of correlation coefficient. By examining the plot presenting correlation between FFR and IMR values, it seems that this relationship was “weaker” in the lower quartiles of IMR values (29).

However, to the best of our knowledge, no previous trials have investigated the impact of TMZ on functional microvascular indices (FFR, IMR and CFR) after elective PCI.

The effects of drugs on microcirculation have been investigated in the setting of elective PCI. In the ProMicro trial intracoronary enalaprilat given during PCI significantly reduced IMR and increased CFR in stented arteries and reduced levels of high- sensitive troponin after procedure (16). This may be the result of increased availability of bradykinin and nitric oxide induced by this drug. Similar effect on coronary microvasculature after PCI has been observed with nicorandil. Given before PCI, nicorandil reduced IMR and cardiac troponins release after elective PCI. Possible explanations for this effect include suppression of Na+/Ca2+ exchange and the reduction of calcium in the myocardium by activation and reduced inflammatory reactions by suppressing neutrophil activation (28). Finally, it has been shown that prasugrel may have protective effect on microvascular circulation after elective PCI by reducing platelet activity and micro thrombosis over less potent PY12 inhibitor clopidogrel and that colchicine given before PCI might also reduce microvascular injury by reducing inflammation (30, 31).

The possible mechanism of TMZ effect on microcirculation in the settings of PCI might be related to beneficial effects on myocardial metabolism that enhance local vasodilation and clearance of microthrombi induced by distal embolization. This can further decrease IMR values and lower troponin release after PCI (7, 9, 10). The beneficial effects of a drug in terms of microcirculatory functional improvement by decreasing IMR in PCI settings might be a method to assess whether it can be further explored as a treatment for microvascular angina in patients with no obstructive coronary artery disease (32). Whether TMZ should be prescribed long term after PCI in specific patients with proven disturbance of microcirculation remains an open question. In a large randomized ATPCI study, that included over 6,000 patients followed for almost four years, adding TMZ to optimal medical therapy after PCI did not affect occurrence of composite endpoint consisting of cardiac death, hospitalization for cardiac reasons and worsening of angina. In this landmark trial patients didn’t undergo invasive coronary physiology and coronary microcirculation was not investigated. The PCI procedure had to be completed and successful, as perceived by the operator, while no further revascularization was planned. The patients in the study reaching angina endpoint were not evaluated for objective angina assessment in terms of specific questionnaires or stress tests (33). Our study was “a proof of concept” trial that TMZ affects microcirculation and since angina after PCI is a multifactorial disease, the results of our study cannot deliver specific recommendation regarding continuation of TMZ after successful PCI even in patients with high IMR value after PCI. Highly selective study that would include patients with documented microvascular dysfunction might explore benefits of adding TMZ to standard therapy after PCI during long term follow up.

### Limitations of the study

Our study has two potential limitations. The study sample is relatively small and that can cause an error in results interpretation. Plaque composition heterogeneity has not been accounted for and that can influence quantity and composition of debris causing distal embolization and dysfunction of microcirculation. However, we believe that these limitations did not affect overall quality of the study data.

## Conclusion

Use of trimetazidine prior to elective PCI increases postprocedural FFR values and reduces microvascular dysfunction by lowering postprocedural IMR values when compared to standard therapy. Trimetazidine might be further investigated as potential treatment option for patients with INOCA.

## Data Availability

The original contributions presented in the study are included in the article, further inquiries can be directed to the corresponding author.
